# Drug repurposing with network reinforcement

**DOI:** 10.1186/s12859-019-2858-6

**Published:** 2019-07-24

**Authors:** Yonghyun Nam, Myungjun Kim, Hang-Seok Chang, Hyunjung Shin

**Affiliations:** 1 Department of Industrial Engineering, Ajou University, 206, World cup-ro, Yeongtong-gu, Suwon-si, Gyeonggi-do 16499 Republic of Korea; 2 Department of Surgery, Thyroid Cancer Center, Gangnam Severance Hospital, Institute of Refractory Thyroid Cancer, Yonsei University College of Medicine, 211 Eonjuro, Gangnam-gu, Seoul, 06273 Republic of Korea

**Keywords:** Drug repurposing, Drug scoring, Semi-supervised learning, Network reinforcement

## Abstract

**Background:**

Drug repurposing has been motivated to ameliorate low probability of success in drug discovery. For the recent decade, many in silico attempts have received primary attention as a first step to alleviate the high cost and longevity. Such study has taken benefits of abundance, variety, and easy accessibility of pharmaceutical and biomedical data. Utilizing the research friendly environment, in this study, we propose a network-based machine learning algorithm for drug repurposing. Particularly, we show a framework on how to construct a drug network, and how to strengthen the network by employing multiple/heterogeneous types of data.

**Results:**

The proposed method consists of three steps. First, we construct a drug network from drug-target protein information. Then, the drug network is reinforced by utilizing drug-drug interaction knowledge on bioactivity and/or medication from literature databases. Through the enhancement, the number of connected nodes and the number of edges between them become more abundant and informative, which can lead to a higher probability of success of in silico drug repurposing. The enhanced network recommends candidate drugs for repurposing through drug scoring. The scoring process utilizes graph-based semi-supervised learning to determine the priority of recommendations.

**Conclusions:**

The drug network is reinforced in terms of the coverage and connections of drugs: the drug coverage increases from 4738 to 5442, and the drug-drug associations as well from 808,752 to 982,361. Along with the network enhancement, drug recommendation becomes more reliable: AUC of 0.89 was achieved lifted from 0.79. For typical cases, 11 recommended drugs were shown for vascular dementia: amantadine, conotoxin GV, tenocyclidine, cycloeucine, etc.

**Electronic supplementary material:**

The online version of this article (10.1186/s12859-019-2858-6) contains supplementary material, which is available to authorized users.

## Background

Drug development and trials in animals and humans is a long and costly process. In general, the whole process of de novo drug discovery takes 10 to 17 years for development with the cost rising from 300 to 600 million dollars [1]. To overcome the de facto difficulty, drug repurposing (or repositioning) has received much attention in recent years [2, 3]. Drug repurposing finds new indicators in already-approved drugs that could be used for treating other diseases. Sometimes, side-effect of a certain drug gives a hint on its repurposing to other diseases. Botulinum toxin and sildenafil (a.k.a., Viagra) are well known as such cases. There are three channels of approaches, in vitro, in vivo, and in silico, in drug repurposing. Compared to de novo drug discovery, in vitro, and in vivo approaches have advantage of reducing the development time, down to 3 to 12 years but they are only available with a good years of expertise on clinical and pharmaceutical domain [4–6]. To find new indicators for drugs, in silico approaches, on the other hand, attempt computational dry-runs that simulate and search all the possible combinations of drugs and diseases from databases which have been more available nowadays. In fact, one channel is not an alternative to others but rather complementary to each other, so it is accepted as a packaged pipeline for drug repurposing, in silico prior to in vitro and in vivo [7].

Up to date, there have been numerous studies for in silico drug repurposing. There are two streams of those studies: drug-centric and disease-centric. The former performs repurposing in pharmaceutical aspect concerning chemical structures of drugs (or compounds) and usages of medication [8–10]. Lamb et al. (2006) used information on molecule movements of components of drugs, Keiser et al. (2009) examined chemical structure and target protein information of drugs, and Chang et al. (2010) utilized both tissue localization and gene expression patterns for analysis. On the other hand, disease-centric approaches repurpose drugs in pathological and clinical aspects, by obtaining knowledge from disease-gene or disease-protein relations [11, 12]. Campillos et al. (2008) predicted new drug targets by using similarities between diseases based on possible side-effects appearing from drugs. Meanwhile, Chiang et al. (2009) suggested a drug repositioning approach under the assumption that if two diseases share few, but similar, number of treatments, then there is a room for repositioning. Despite the disparate views, whether it is drug-centric or disease-centric, the main idea of disclosure for new usages of existing drugs is similarity between drugs or between diseases.

One effective way to describe similarities between entities is network representation of nodes and edges. Given a set of drugs, drugs are the nodes and drug-drug similarities (or associations in a broader concept) are represented on the edges [13]. In these days, there are a number of pharmaceutical and biomedical data sources that can be employed for calculating similarities between drugs. Also, there have been constant advances in machine learning algorithms that can represent heterogeneous types of data as a form of network and draw a good inference from the network structure. In a word, a bunch of data sources and tools of high quality are more available than ever. These environmental advantages can lead in silico drug repurposing to a more reliable and realistic approach.

**Fig. 1:**
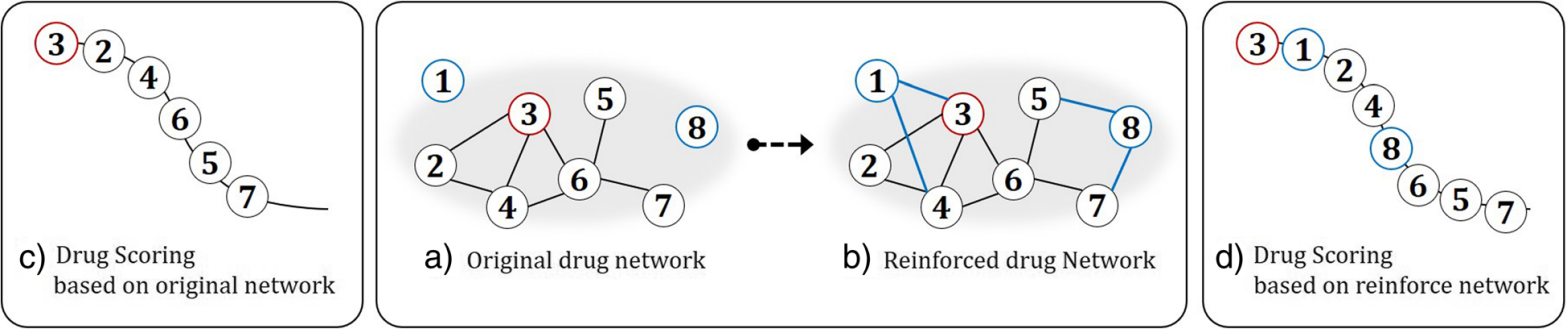
The main idea of drug repurposing with network reinforcement: Red circle represents the originally known drugs, and blue circle represents isolated (disconnected) drugs from the drug network. The center panel shows the drug network constructed by drug-protein association and its reinforced network with additional information. Left and right panel show the scoring results according to (a) and (b), respectively

In this study, we propose an algorithm performing drug repositioning based on a network of drugs. We start from presenting how to construct a drug network, and how to evolve or strengthen it, taking benefits of multiple/heterogeneous types of data. For the process of constructing a drug network, we first construct a network with information on drug-target protein. The drug network, however, can be sparse (mostly unconnected) due to unidentified target proteins for drugs or insufficient knowledge of the drugs. In drug repurposing perspective, if a drug is undefined in terms of interactions with other drugs, we cannot obtain sufficient pharmaceutical evidences for repurposing existing drugs to other disease. In network-modeling perspective, a sparse network may not provide satisfactory inference or performance because of deficiency in flow of information via edges [14–17]. To overcome the difficulty, we enhance the original network to make up the lack of connections by utilizing complementary data sources. Complementary Linkage with Anchoring and Scoring (CLASH) is employed [15]. It is an algorithm that complements a network by using external knowledge while preserving original information of the network. In this study, the algorithm is applied to our drug network and further extended by including more data sources. In the network, drug-protein relation become a base source, and additional information on bioactivity and medication from PubChem and PubMed strengthen the network capability by complementing insufficient connections of network. On the resulting drug network, scoring is run for finding candidate drugs for repurposing. Scores endowed to drugs provide priority ranks for recommendation. At last, drugs in top-tier become the candidates of repurposing. Figure 1 describes the concept of reinforcement for drug network. In the center, there are two networks: (a) original network, (b) Reinforced network. Network (a) is constructed only with drug-target protein association. Network (b) is the reinforced network with additional information. In network (a), two drugs 1 and 8 (marked as blue) are disconnected in the graph. If drug-target protein association is not identified or if some of drugs do not have shared target proteins, they are isolated in the graph. In such case, if we want to find similar drugs or compounds with drug 3 (marked as red), we cannot measure the scores for drug 1 and 5 (See Fig. 1(c)). However, if the network is reinforced with additional information, as in network (b), we can calculate the scores for all drugs including drug 1 and 8 (as shown on the right side of the Fig. 1). With the reinforced drug network, it leads to a higher probability of success of in silico drug repurposing.

More details of the how to construct drug network and how to reinforce/complement the network is described in the methods section. In the results, we provide experimental results on validity and utility for drug scoring. Also, we present and discuss the typical results for drug scoring with vascular dementia.

## Results

### Experimental settings

The proposed method was applied to 5442 drugs and 1938 diseases. A list of 5442 drugs and 1938 diseases was obtained from PubChem and Medical Subject Headings (MeSH) of the National Library of Medicine. For relation data, we used 91,450 drug-target protein information and 61,794 disease-drug association. For the original drug network, 4738 drugs (out of 5442) and 19,465 target protein were used, leaving other 704 drugs, which had no drug-target protein information, unconnected. The edges were calculated with cosine similarity between 19,465-dimensional drug vectors. To enhance the drug network, 77,729 drug-drug interaction knowledge from PubChem and PubMed were used as external data sources. These external knowledges include drug interactions (or compound relation) shown in literature, medications, and bio-activities. From this point onward, we define the original drug network as a network constructed with drug-target protein information and the enhanced network as a network complemented with external sources of information. To validate performance of drug network, we utilized disease-drug associations. Table 1 summarizes the data and its source used for our experiment.

**Table 1:** List of data sources to construct/enhance drug networks

Description	# of data	Sources
Drug	5442	PubChem (https://pubchem.ncbi.nlm.nih.gov/)
Disease	1938	MeSH (https://www.ncbi.nlm.nih.gov/mesh)
Protein	19,465	Entrez Gene (https://www.ncbi.nlm.nih.gov/gene)
Drug-Protein	91,450	PubChem (https://pubchem.ncbi.nlm.nih.gov/) PubMed (https://www.ncbi.nlm.nih.gov/pubmed/) PharmGKB (https://www.pharmgkb.org/) DrugBank (https://www.drugbank.ca/) T3DB (http://www.t3db.ca/) TTD (http://bidd.nus.edu.sg/group/cjttd/) CTD (http://ctdbase.org/) DCDB (10.1093/database/bau124)
Drug-Drug Interaction	77,729	
Disease-Drug Association	61,794	

**Fig. 2:**
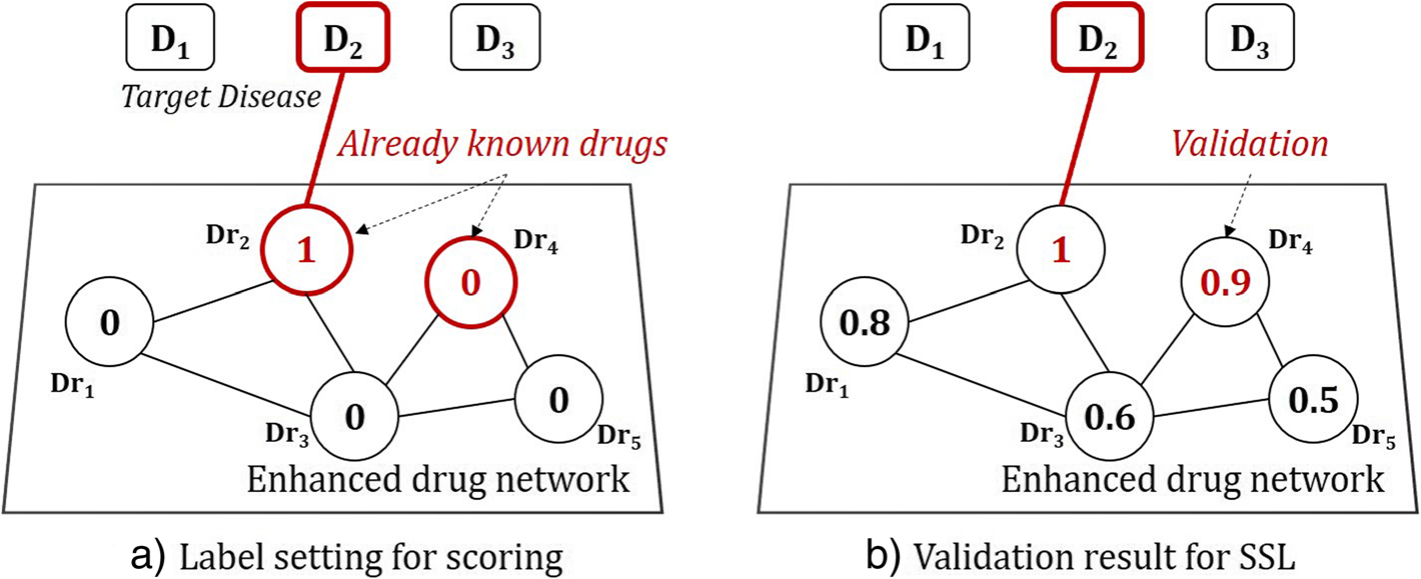
Experimental setting for measuring prediction performance: (**a**) a drug network constructed with five drugs with target disease D_2_. If dr_2_ and dr_4_ are already in use for treating D_2_, we randomly select one and assign label ‘1’ (**b**) validation of the SSL result for non-selected (but already in use) dr_4_

To verify the performance of the proposed drug repurposing method, we applied it to prediction of drugs for treating a certain. The performance comparison was conducted on three networks: original network, data fusion network, enhanced network. Original network was constructed by 4738 drugs with drug-target protein information. To construct data fusion network, it was simply integrated with original network and drug-drug interaction network. Data fusion network is simply a combination of two networks. The enhanced network is a proposed method that selectively uses drug-drug interaction for external sources based on original network. To obtain predictive outputs for drugs, we used graph based semi-supervised learning algorithm. Given a target disease, SSL provides scores for all drugs. For experiment setting, we first selected a target disease and gave label ‘1’. Then if we know 20% of drugs already in use for the target disease in a priori, we randomly assigned 20% drugs associated with the target disease with label ‘1’s and gave ‘0’s to remaining drugs (See Fig. 2). The experiment was carried out with 5-fold cross validation and the whole experiment was repeated 10 times.

### Results for comparative performance

Table 2 shows the overall properties of three networks. Original network was constructed by 4738 drugs with already existing information. Data fusion network is simply a combination of two networks. For the enhanced network, the number of drugs increases from 4738 to 5442 and the drug-drug associations from 808,752 to 982,361. The density of original network is 5.46% while that of the enhanced network is 6.64%. Network density is calculated by actual connection over potential connections. We achieved an improvement of density with an increase by 21.47%. The density of data fusion network is larger than that of the enhanced network. In case of enhanced network, the newly connected edge is selectively used. This is a selective selection of information that does not degrade performance after reinforcement.

**Table 2:** Overall properties of constructed networks

	Original network (Reference)	Data fusion network (Simple integration)	Enhanced network (Proposed method)
Number of nodes (drugs)	4738	5442	5442
Number of edges	808,752	1,054,324	982,361
Density of networks	5.46%	7.12%	6.64%

**Fig. 3:**
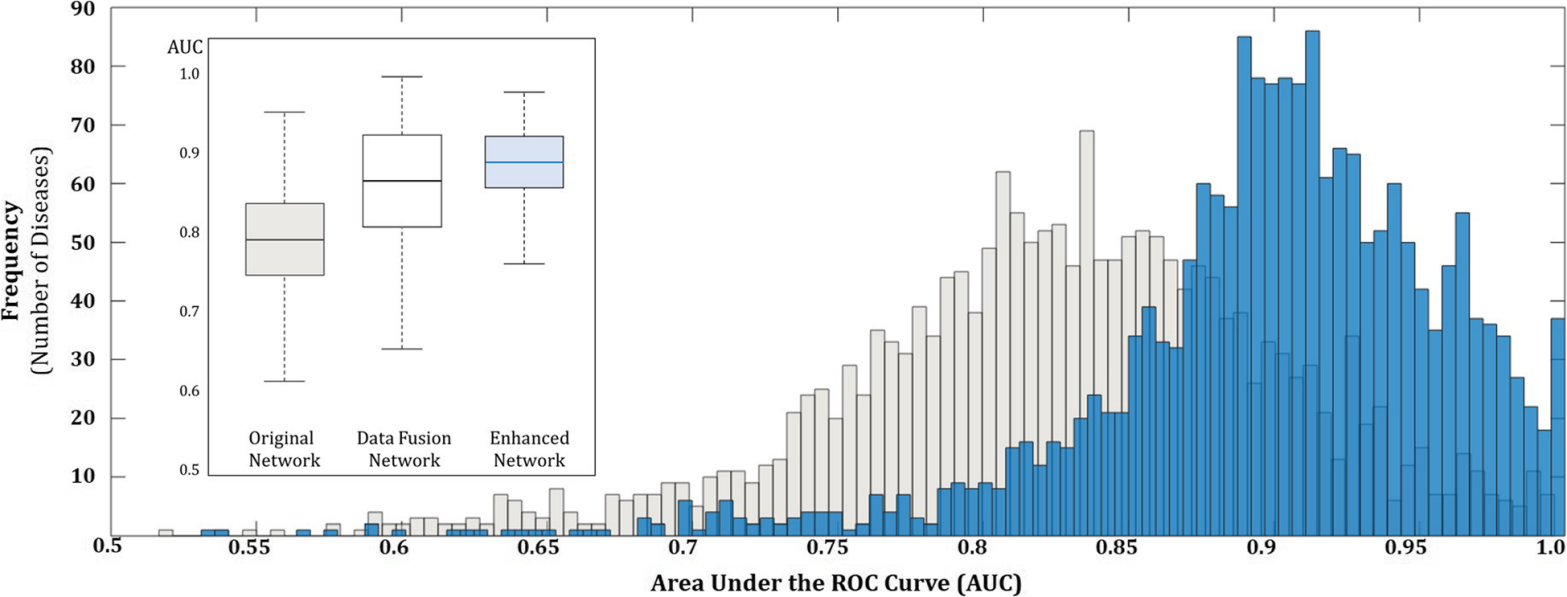
Performance improvement of the enhanced network: the gray and blue bar represents histogram of AUC for the original and enhanced network, respectively. The histogram is shifted towards right for the enhanced network. In addition, the inserts summarize overall average AUC with deviation. The *p*-value for statistical tests for pairwise comparison between original network-reinforce network and data fusion network-reinforce network are 0.0003 and 0.0005, respectively

The validation results are summarized in Fig. 3. Figure 3 illustrates a comparison of performance of the original network and the enhanced network. The network performance is measured by the Area under the ROC curve (AUC) [18]. In the figure, the distribution of AUC for 1938 diseases in both original and enhanced network is presented. The x-axis represents section by section AUC and y-axis represent frequency of target disease belonging in the range. The figure shows that the distribution from the proposed enhanced network is shifted towards right compared to that of the original network. This demonstrates that the network performance of the enhanced network is outstanding compared to the original network. Moreover, from the box plot, which describes the overall AUC, the proposed method improves the performance up to Avg. 0.89 (lifted from 0.79). As shown in the box plot, the average of the AUC for data fusion network was 0.85. The performance difference between data fusion and enhanced network may not appear to be significant. However, through the deviation, the enhanced network is more robust in the prediction results than the other two networks. The *p*-value for statistical tests for pairwise comparison between original network-reinforce network and data fusion network-reinforce network are 0.0003 and 0.0005, respectively.

**Fig. 4:**
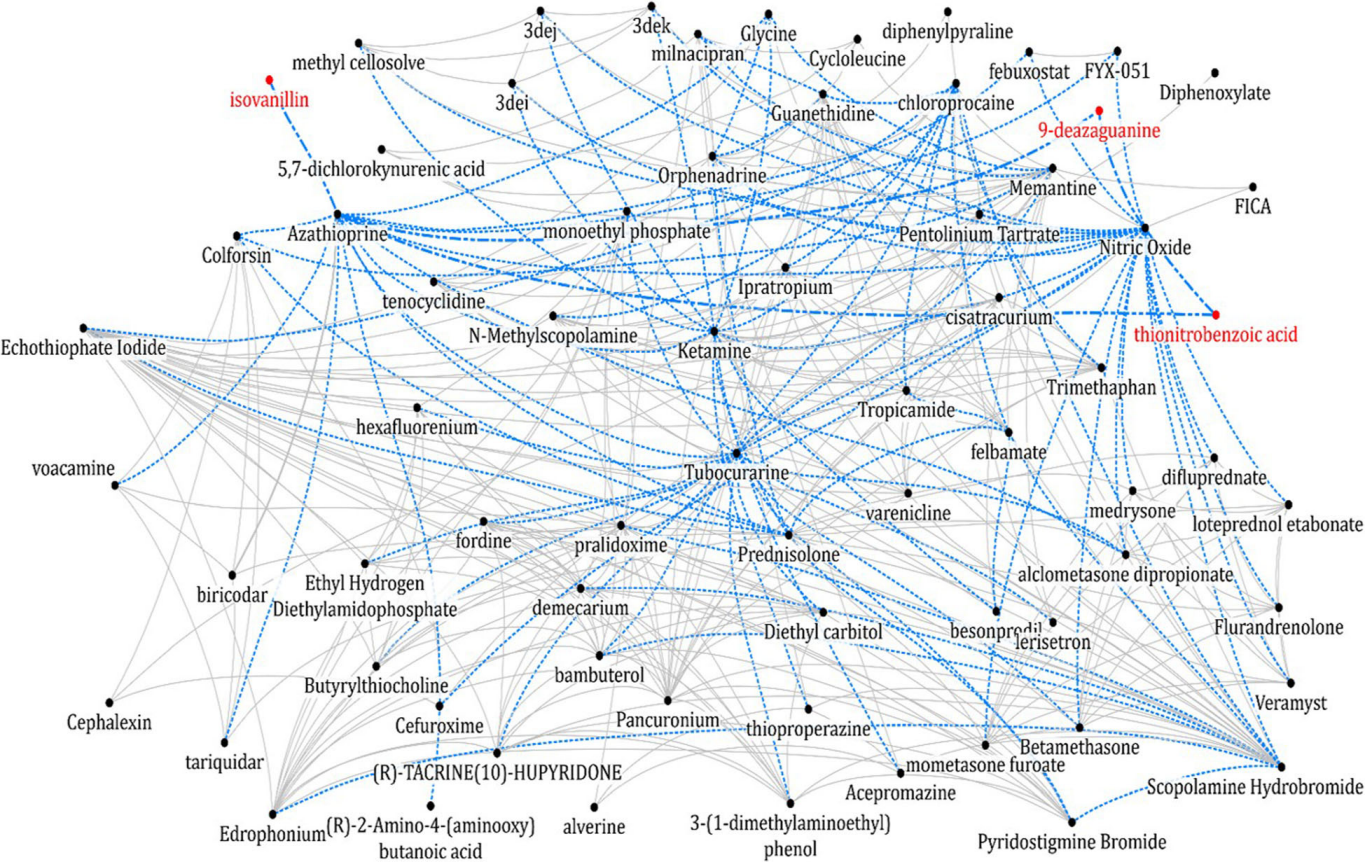
A snapshot of the enhanced drug network: the solid lines represent original connections with information on shared target protein, and the dotted lines represent newly connected edges using CLASH. Red circles represent orphan drugs but linked to the network by the proposed method. For better readability, the network is simplified. More detailed one is provided in Additional file 1

To demonstrate the network reinforcement, we present a sub-graph of the enhanced network. Figure 4 depicts a snapshot of a small sub-graph of 70 drugs drawn from the proposed method. In the network, each node represents a drug or a compound. The black circles represent originally connected drugs and the red circles represent isolated ones that do not have shared target proteins with other drugs or do not contain sufficient target-protein information. The solid and dotted lines describe original connection with information on shared target protein and newly connected edges using CLASH, respectively. Three drugs were originally unconnected to the network due to lack of shared target protein. By applying the proposed method, unconnected nodes were connected: network density increased from 11.18 to 14.87%; the drug coverage increases from 67 to 70, and the connections between drugs from 270 to 359.

## Discussion

To show utility of drug scoring, we applied the *in-silico* repurposing method to dementia disorder. Dementia refers to a condition in which a person has multiple cognitive impairments and mental disorders that severely affect daily life. Symptoms of dementia include loss or changes in memory, problems with abstract thinking, disorientation to time or places, and drastic changes in personality [19, 20]. Among various types of dementia, more than 80% of patients have Alzheimer’s disease and vascular dementia, which have cranial nerve lesion. Since the pathogenesis of these diseases have not been completely identified, drugs used in the clinical field only aid in preventing or relieving symptoms of the diseases. Therefore, drug discovery for dementia treatment is an important issue in the pharmaceutical industry. Among various dementia disorders, we demonstrate typical results for vascular dementia and Parkinson diseases. Vascular dementia is a decline in thinking skills caused by conditions that hinder blood flow to the brain, thereby depriving brain cells of vital oxygen and nutrients. In contrast to Alzheimer’s disease, vascular dementia accompanies neurological symptoms such as hemiplegia, facial palsy, blindness, visual field defects, gait disturbance, etc. [21, 22].

**Fig. 5:**
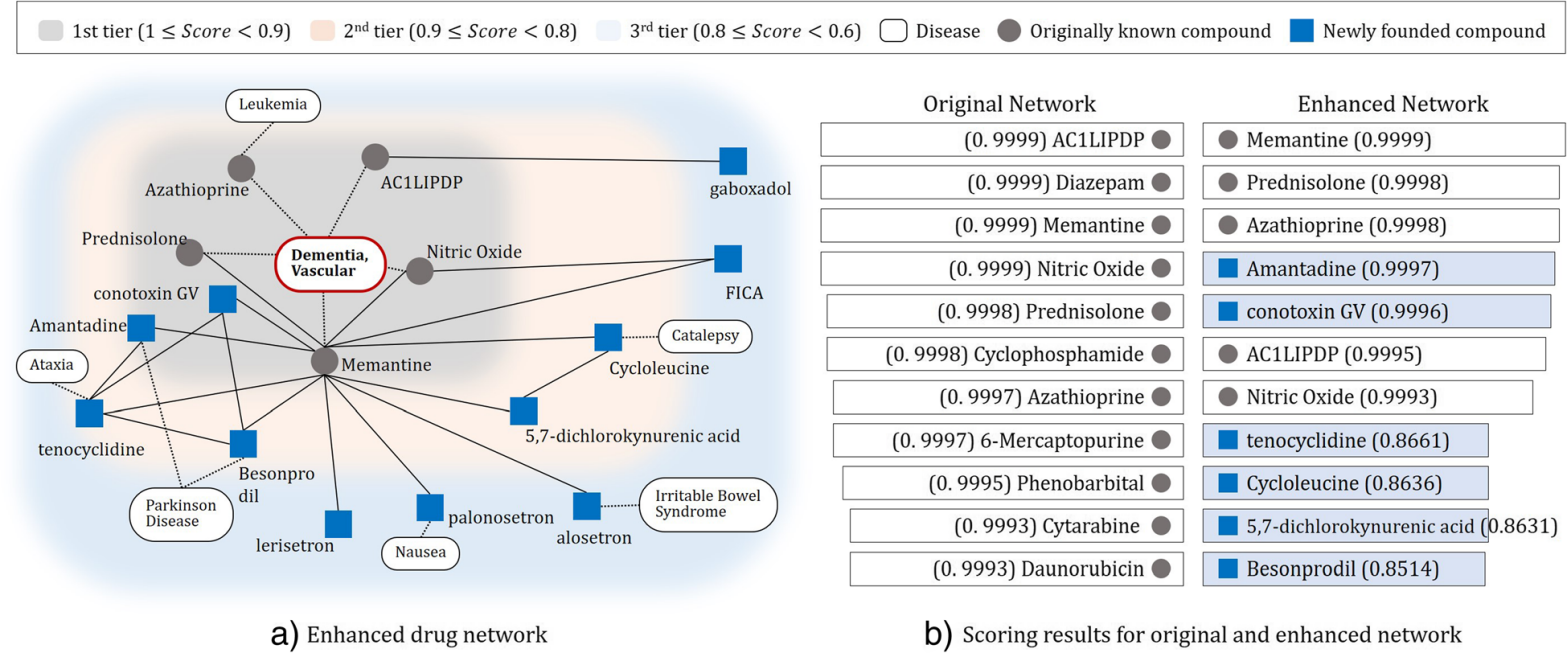
Enhanced drug network focused on vascular dementia: (**a**) The network is divided into three regions, in which the 1st tier region around the center consist of drugs with score value greater than 0.9. (**b**) Left panel shows the drug scoring results using original network. Right panel shows the proposed scoring results using enhanced network

**Fig. 6:**
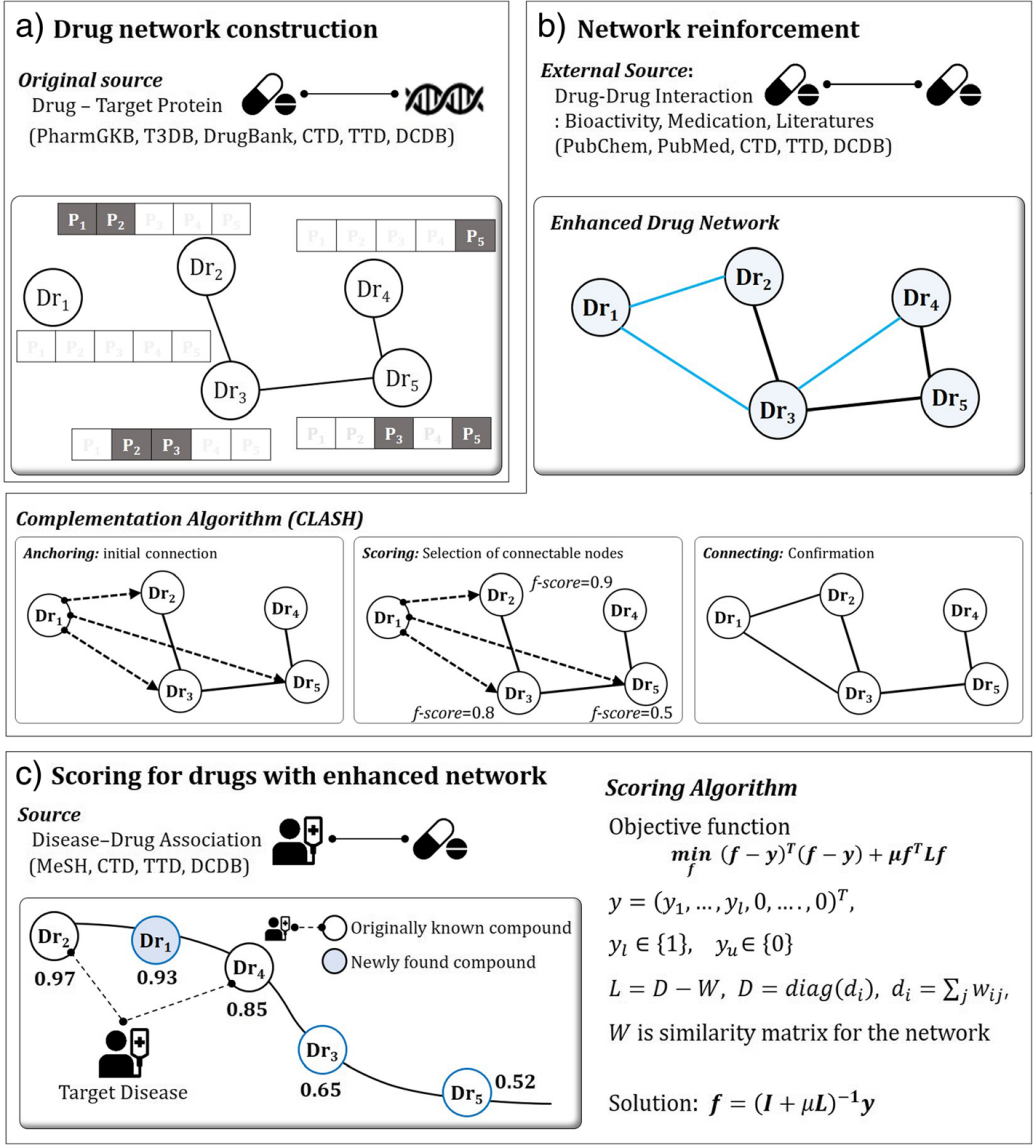
Drug repurposing with network reinforcement: The proposed method has three steps; (**a**) drug network construction based on drug-target protein, (**b**) network reinforcement with external knowledge such as bioactivity, medications, etc., and (**c**) drug scoring with SSL for a specific disease based on the enhanced network

Figure 6 depicts the drug network focused on vascular dementia. On the graph, the drugs that have potential for treating vascular dementia are positioned so that it reflects the result of drug scoring: the closer the drug towards vascular dementia, the higher of its possibility of treatment. In the Fig. 5(a), the gray circles represent drugs or compounds that are already in use for vascular dementia and the blue circles represent candidate drugs that are newly found with drug scoring. The network is divided into three regions, in which the 1st tier region around the center consist of drugs with score value greater than 0.9. Among six drugs in 1st tier region, memantine, prednisolone, azathioprine, AC1LIPDP, and nitric oxide are already in use for treating vascular dementia. Figure 5(b) describes the difference of scoring results according to the types of network. Left panel show the results of drug scoring using original network and the right panel is the enhanced network. In the original network, only originally known compounds had a high score. On the other hand, the enhanced network not only shows the already used drug but also the new candidate drug. This result shows that the network includes drug-drug interaction information obtained through literature as well as drug-target protein information via network reinforcement.

Table 3 shows traits and validations for recommended drugs. For exemplary cases, we examined Amantadine and Conotoxin GV, which are top two recommended drugs with high score values. In the enhanced network, Amantadine was connected with memantine based on drug-drug interaction information from bioactivity and literature. Usually, Amantadine is an antiviral used in the prophy-lactic or symptomatic treatment of influenza but is also known as an antiparkinsonian agent to treat extrapyramidal reactions, and for postherpetic neuralgia. Unlike Amantadine, Conotoxin GV was already connected in the original network. While having low score in the original network, Conotoxin GV had high score value with newly connected edges from the enhanced network. Therefore, we recommend Conotoxin GV as a newly found candidate drug for repurposing. For some verification, Conotoxin GV is known to act on N-methyl D-aspartate acid receptor (NMDA receptor), where NMDA receptor is responsible for memory and learning functions in the brain. Other nine candidate drugs are shown in Fig. 5(a). The implemented results can be used to identify the priorities of candidate drugs with potential of dementia treatment. This can serve as a screening tools for in vivo or in vitro drug discovery.

**Table 3:** Traits and validation of the recommended drugs

Drug (Compound)	Amantadine	Conotoxin GV	Tenocyclidine	Cycloleucine
Remarks for validation	***Bioactivity/Medication:*** Amantadine can be used as an anti-parkinsonian agent, to treat extrapyramidal reactions, and for postherpetic neuralgia.	***Enhanced Network:*** Conotoxin GV is known to act on N-methyl D-aspartate acid receptor (NMDA receptor)	***Sharing target protein:*** Tenocyclidine share four target proteins with memantine: *Alpha-7 nicotinic cholinergic receptor subunit*, *Glutamate receptor ionotropic, NMDA 2A / 2B / 3A*	***Sharing target protein:*** Cycloeucine share one target protein with memantine: *Glutamate receptor ionotropic, NMD1*; PMID10051153, 7,773,540

## Conclusion

In this study, we propose an enhanced drug network that could be utilized for in silico drug repurposing. The method consists of three steps. First, we constructed a drug network based on drug-target protein information. Second, in order to overcome inherent difficulty, sparseness of the network, we enhanced the network with various external sources. To perform this, a network complementation algorithm, CLASH was employed. The algorithm succeeded in connecting 704 drugs previously isolated. Finally, to the resulting network, we applied graph based semi-supervised learning to score drugs. The top ranked drugs were recommended as candidates for repurposing. We validated the method by applying it to 5442 drugs and 1938 diseases. The AUC performance, we obtained is 0.89, a significant lift from 0.79. We showed the typical cases of vascular dementia and Parkinson disease to present exemplar utility of the proposed method: 11 candidate drugs were recommended and validated for repurposing. Not limited to the dementia disorders, the proposed method can be generally extended to other diseases.

This research has novelty in the following aspects. The proposed method not only utilizes drug-target protein information, but also employs latest research information on drug-drug interactions. Thus, it is regarded as effective for finding new indicators in drugs. One of advantages of the proposed method lies in that we can instantly and easily update the candidate drug list with recent knowledge. And, the network-based approach can ease us to read and comprehend intricate associations between drugs and/or diseases. We can further improve the proposed network by expanding scopes of complementary knowledge sources to drug side-effects and drug chemical structures, etc. Also, we did not consider negative correlation of newly added connections with antagonistic properties. It would be interesting to consider such cases and develop more sophisticated algorithms. On the other hand, we can personalize the network by enhancing it with patient-specific genetic information. This personalized network will be a strong tool for the era of precision medicine.

## Methods

The drug repurposing method that we propose in this study consists of three steps: drug network construction, drug network enhancement, and drug scoring. In the first step, we construct a drug network by calculating similarity between drugs based on shared drug-target protein. However, since not all relationships between drugs can be identified with shared target proteins, the network can be very sparse. With such sparseness, the number of drugs for repurposing can be very limited. To circumvent the difficulty, we enhance the drug network by increasing the number of nodes and edges. We apply a network complementation algorithm, CLASH. This increases the density of network leading to higher and more stabilized performance: the latest possible information from PubChem and PubMed is reflected to the network by connecting two drugs that previously have no connection. Finally, a candidate drug chosen out of approved ones is applied as a new usage to a certain disease. With the enhanced drug network, we use a scoring algorithm that assigns scores to drugs when a specific disease is given. Disease-drug association is used to pick the related drugs to the disease. Through the procedure, the priority of drugs is determined and the ones in the top-tier are recommended as candidates for repurposing drugs.

### Drug network construction

Drug network is a graph, *G* = (*V*, *W*), that represents connection between drugs (or compounds) with nodes and edges. In a drug network, a node denotes drug and an edge denotes a value obtained by calculating similarity between two drugs based on their shared target proteins [13]. More specifically, a drug vector has n-dimensional protein vector, and the similarity between two drugs are calculated with cosine similarity between drug vectors (See Fig. 6(a)). On the graph, similarity between two drugs are assigned with a weight value on the edge and higher of its value implies higher relation between two drugs.

### Network reinforcement

The complementation process can be applied to two cases. First, drugs with no information on target proteins are unconnected with other drugs in the network. This corresponds to *dr*_1_ in Fig. 6(a). Second, two drugs can be unconnected if they do not have shared target proteins. This corresponds to *dr*_2_ and *dr*_4_ in Fig. 6(a). To enhance the network, we apply a complementation algorithm, CLASH, which reinforces a network with external source while preserving the original source of information [15]. The basic assumption of the complementary process is to maintain the properties of the original network. The algorithm consists of anchoring step for initializing connections on nodes, scoring step for determining priority of connection, and connecting step for confirming the connection. The algorithm stops when there are no more possible nodes to connect. With CLASH, we first initialize connections with anchoring step, then finalize complementation in step by step manner to prevent the performance of the original network. For CLASH, not all external source of information is used. The algorithm only accepts new sources that does not harm the original knowledge of information. Furthermore, if two nodes are connected in the original network, no external source is used to reinforce the network. The following describes an exemplary process of network reinforcement.

- **Anchoring Step:** When we have *n* drugs, we construct a drug network *G* = (*V*, *W*). In the graph, we can define the set of original drugs *S*_*O*_ = {*v*_*i*_| *v*_*i*_ ∈ *V*, *i* = 1, … , *n*}, set of connected drugs *S*_*c*_ = {*v*_*i*_| *v*_*i*_, *v*_*j*_ ∈ *S*_*O*_, ∃_*j*_ *v*_*i*_~*v*_*j*_}, and set of isolated (disconnected) drugs *S*_*D*_ = {*v*_*i*_| *v*_*i*_, *v*_*j*_ ∈ *S*_*O*_, ∀_*j*_ *v*_*i*_ ≁ *v*_*j*_}. At the anchoring step, complementary algorithm builds the pendant edges (virtual edges) between an isolated drug and connected drugs from available external sources. Then we have a anchoring set of *v*_*i*_, ${S}_A^i=\left\{{v}_j|{v}_i\in {S}_D,{v}_j\in {S}_c,{v}_i\sim {v}_j\right\}$ where *v*_*i*_~*v*_*j*_ is identified from available external sources. And we have a validation set of *v*_*i*_, ${S}_V^i={S}_c\backslash {S}_A^i$. In Fig. 2(b), *dr*_*1*_ is initially anchored to {*dr*_2_, *dr*_3_, *dr*_5_} based on external source of information. The anchoring set *S*_*A*_ = {*dr*_2_, *dr*_3_, *dr*_5_} remains connectable drugs. At this point, edges between isolated drugs *S*_*D*_ and connected drugs *S*_*C*_ remain virtually connected.
- **Scoring Step:** The scoring step allows a disconnected drug to select connectable drugs from anchoring set *S*_*A*_. To order the connectable drugs, we apply graph-based semi-supervised learning (SSL). (more details on SSL is described in drug scoring section) In a virtually connected graph, SSL calculate the scores ***f*** = {*f*_1_, *f*_2_, …*f*_*n*_}^T^ when a disconnected drug is given. In Fig. 6(b), the scores for anchored drugs {*dr*_2_, *dr*_3_, *dr*_5_} have the score values {0.9, 0.8, 0.5}, respectively.
- **Connecting/Stopping Step:** This is the confirmation step for allowing isolated (disconnected) drugs to be connected to the graph based on scoring results. The order of connection is determined by scores on anchored drugs. The connection step sorts *f*_1_, … , *f*_*n*_ by descending order of scores f and it connects *v*_*i*_ to *v*_*j*_ ’s which have the largest scores. Complementary algorithm check network performance by using validation set *S*_*V*_ to preserve the network’s property which means that the connecting step prevents the degradation of network’s performance. The algorithm stops when there are no more unconnected nodes, no more external data, or the performance of the network decreases. In Fig. 6(b), three connections are possible with available external source but dr1 and dr5 are not connected due to the criterion of finalizing the connection. The degree of harmfulness of external source is determined by measuring change in performance of the network.

### Drug scoring for repurposing

Given a specific disease, the enhanced network recommends candidate drugs for repurposing through drug scoring. The scoring process determines the priority of recommendations and utilizes one of machine learning algorithm, graph-based Semi-Supervised Learning (SSL). Since drug-drug interactions are sparsely known, most machine learning algorithms cannot perform well. On the other hand, SSL can predict unlabeled nodes by using both labeled and unlabeled nodes. If we let the similarity matrix of the constructed drug network as ***W***, SSL uses the graph Laplacian matrix, ***L***, and yields score ***f*** from the following functional:

$$ \boldsymbol{f}={\left(\boldsymbol{I}+\mu \boldsymbol{L}\right)}^{-1}\boldsymbol{y} $$

where ***f*** = (*f*_1_,  … , *f*_*n*_)^*T*^*,* ***y*** = (0,  … , 0, *y*_*i*_ = 1, 0,  … , 0)^*T*^*,* and *μ* is a user-specified parameter. Here, the graph Laplacian ***L*** is defined as ***L = D − W*** where ***D =***  *diag* (*d*_*i*_), and ${d}_i=\sum \limits_j{w}_{ij}$ For more details see [23]. The larger score of ***f*** for a drug implies higher possibility of treating the given disease. In Fig. 6(c), we recommend drugs in the top-tier, in the order of ***f***-score, as candidates for repurposing.

## Additional file

Additional file 1: **Figure S1.** A snapshot of the enhanced network with 150 drugs: the solid lines represent original connections with information on shared target protein, and the dotted lines represent newly connected edges using CLASH. Red circles represent orphan drugs but linked to the network by the proposed method. (PDF 751 kb)

## Abbreviations

CTD: Candidate Target Gene
MeSH: The Medical Subject Headings
PharmGKB: The Pharmacogenomics Knowledge Base
TTD: Therapeutic Target Database
