# Supplementary material for: Drug repurposing with network reinforcement
Source: BMC Bioinformatics. 2019 Jul 24;20(Suppl 13):383. doi: 10.1186/s12859-019-2858-6 (PMC6651901; doi:10.1186/s12859-019-2858-6)
Supplement: Supplementary file 1 — Figure S1. A snapshot of the enhanced network with 150 drugs: the solid lines represent original connections with information on shared target protein, and the dotted lines represent newly connected edges using CLASH. Red circles represent orphan drugs but linked to the network by the proposed method. (PDF 751 kb) [file 12859_2019_2858_MOESM1_ESM.pdf]

[APPENDIX]

Drug Repurposing with Network Reinforcement

Yonghyun Nam[1], Myungjun Kim[1], Hang-Seok Chang[2], Hyunjung Shin [1,*]

[1]Department of Industrial Engineering, Ajou University, 206 World cup-ro, Yeongtong-gu, Suwon, South Korea
[2]Department of Surgery, Thyroid Cancer Center, Gangnam Severance Hospital, Institute of Refractory Thyroid Cancer, Yonsei University College of Medicine, Seoul, Korea

*To whom correspondence should be addressed.

< Supplements >

- **Appendix A: A snapshot of the enhanced drug network with 150 drugs**

Contact: shin@ajou.ac.kr

[*] Corresponding author: shin@ajou.ac.kr

[Appendix A] Figure S1.

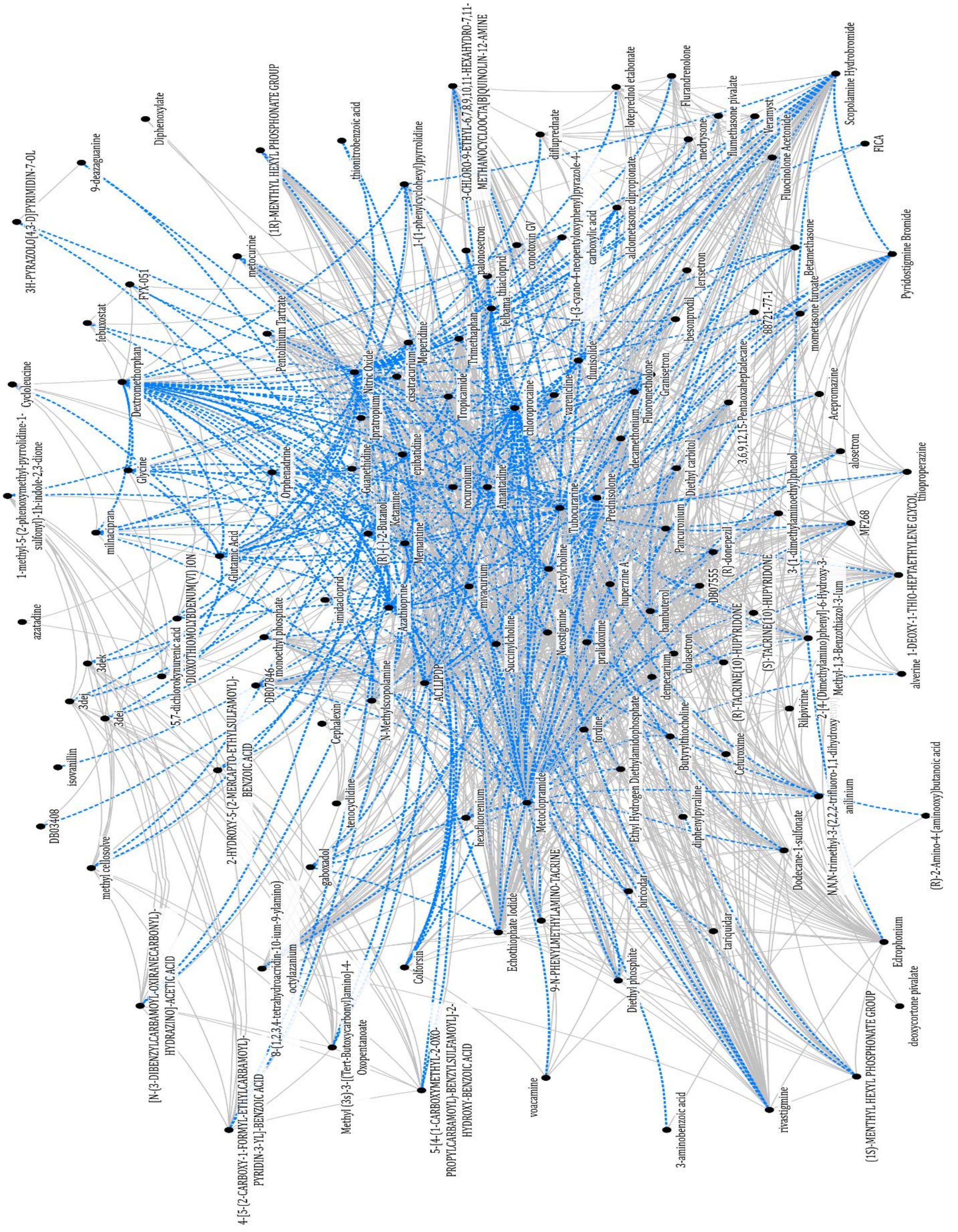

Figure S1. A snapshot of the enhanced network with 150 drugs: the solid lines represent original connections with information on shared target protein, and the dotted lines represent newly connected edges using CLASH. Red circles represent orphan drugs but linked to the network by the proposed method.
